# 非小细胞肺癌新辅助免疫治疗生物标志物研究进展

**DOI:** 10.3779/j.issn.1009-3419.2022.102.04

**Published:** 2022-02-20

**Authors:** 春露 舒, 大兴 朱, 清华 周

**Affiliations:** 1 610041 成都，四川大学华西医院生物治疗国家重点实验室 State Key Laboratory of Biotherapy and Cancer Center, West China Hospital, Sichuan University and Collaborative Innovation Center, Chengdu 610041, China; 2 610041 成都，四川大学华西医院肺癌中心 Lung Cancer Center, West China Hospital, Sichuan University, Chengdu 610041, China

**Keywords:** 肺肿瘤, 免疫检查点抑制剂, 新辅助治疗, 生物标志物, Lung neoplasms, Immune checkpoint inhibitor, Neoadjuvant therapy, Biomarkers

## Abstract

外科手术是早中期非小细胞肺癌（non-small cell lung cancer, NSCLC）患者的主要治疗手段。NSCLC的辅助治疗和新辅助治疗在预防疾病复发和控制远处转移具有重要意义。免疫检查点抑制剂（immune checkpoint inhibitors, ICIs）作为新的药物治疗手段已经在晚期NSCLC患者中带来长期生存获益，并在NSCLC新辅助治疗人群中显示出较高的降期率和病理缓解率。但是如何早期识别、鉴定NSCLC ICIs新辅助治疗的获益人群是当前面临的巨大挑战之一。目前，NSCLC ICIs新辅助的标志物研究尚无定论。因此，我们总结了目前已经披露的新辅助免疫治疗临床试验及相关的生物标记物的研究进展。

## Abstract (en)

Surgery is the standard treatment for resectable non-small cell lung cancer (NSCLC). Neoadjuvant and adjuvant therapy have been widely used for preventing recurrence and metastasis. Immune checkpoint inhibitors (ICIs) have brought long-term survival benefits in advanced NSCLC and showed higher downstage rates and pathological remission in the neoadjuvant setting. Predictive biomarkers are of great significance to identify the beneficiaries of neoadjuvant ICIs. At present, the biomarkers are still inconclusive. We summarized the clinical trials of neoadjuvant immune checkpoint inhibitors that have been disclosed so far, and reviewed the progress of the biomarkers associated with those trials.

肺癌一直是抗肿瘤治疗的重大挑战之一。据全球癌症统计数据^[1]^显示，肺癌的发病率占所有肿瘤的11.4%，死亡率占所有肿瘤的18%。其中，在男性人群中肺癌发病率和死亡率最高，在女性人群中肺癌发病率第二，但死亡率第一。外科手术是根治可切除性肺癌的最重要手段之一^[2]^。然而占肺癌85%左右的非小细胞肺癌（non-small cell lung cancer, NSCLC）中仅有30%-40%可切除。药物的辅助治疗和新辅助治疗旨在预防疾病复发，控制远处转移。而研究^[3, 4]^表明，新辅助化疗和辅助化疗仅可改善5%的NSCLC的5年总生存（overall survival, OS）率，尚未满足临床需求。

免疫检查点抑制剂（immune checkpoint inhibitors, ICIs）作为新的治疗手段，不仅深刻打破了晚期NSCLC治疗格局，而且在早期NSCLC人群中显示出初步疗效。早期临床试验数据^[5]^显示新辅助免疫治疗展现出良好的降期疗效，主要病理缓解（major pathologic response, MPR）率为33%-85%，完全病理缓解（complete pathological response, pCR）率为10%-71%。然而，目前的数据显示新辅助免疫治疗的总体疗效差异较大，尚未明确哪些人群将会从新辅助免疫治疗中获益更多。因此，有效的生物标志物对新辅助治疗人群的选择具有重要意义。

研究数据披露的Ⅲ期临床试验中与免疫治疗疗效相关的标志物大致分为以下四类：①肿瘤细胞相关的生物标志物：包括程序性死亡受体1（programmed cell death ligand 1, PD-L1）表达、肿瘤突变负荷（tumor mutation burden, TMB）、DNA损伤应答（DNA-damage response, DDR）通路、特定基因突变[例如γ-干扰素（interferon γ, IFN-γ）通路、*KRAS*和*STK11*突变]和新抗原；②肿瘤微环境相关的标志物：包括PD-L1表达、肿瘤浸润免疫细胞和免疫状态评分；其中肿瘤浸润免疫细胞包括具有特定表型的免疫细胞（例如，CD4^+^ T细胞、FOXP3^+^ T细胞），免疫组库的多样性[例如，T细胞受体（T cell receptor, TCR）库]；③液体活检相关的生物标志物：包括外周血细胞、循环肿瘤DNA（circulating tumor DNA, ctDNA）和其他分子生物标志物（例如外泌体等）；④宿主相关的生物标志物：包括一般特征（例如，性别、年龄、体脂分布等）、肠道共生体、宿主种系遗传学特征[例如，人类白细胞抗原（human leucocyte antigen, HLA）多样性和其他特定突变等]^[6]^。

在可切除的早期NSCLC人群中生物标志物的探索处于起步阶段。新辅助免疫治疗是寻找生物标志物的理想模式，因其试验周期短且治疗过程包括新辅助治疗期、手术期、辅助治疗期。可以在治疗过程的不同时期提供更多机会观察药物干预后的生物学变化。在早期NSCLC人群的免疫治疗模式中，肿瘤细胞、肿瘤微环境以及外周血相关的生物标志物研究较多。本文将总结当前评估可切除NSCLC患者新辅助免疫治疗的研究，并综述这些研究中相关生物标志物的进展。

## 1 新辅助免疫治疗研究进展

目前全球范围内已经掀起新辅助免疫治疗的研究热潮。据不完全统计，Clinical Trial网站上可以查询到正在进行的临床研究有近40项（表 1，表 2），大部分为Ⅰ期/Ⅱ期研究，另有7项Ⅲ期研究。其中以新辅助免疫单药/免疫联合（CheckMate159、LCMC3、NEOSTAR、NADIM、CheckMate816）等为代表的临床试验结果显示：新辅助免疫治疗对于改善早期NSCLC患者的预后具有良好的应用前景（表 1）。

**表 1:** 已完成或发表的可切除NSCLC新辅助免疫治疗临床试验 Completed or published clinical trials of neoadjuvant immunotherapy in operable NSCLC

Clinical trial	Stage	Drugs	Phase	Surgery rate	MPR	pCR	Major biomarker
NEOSTAR	Ⅰ-Ⅲa	Nivolumab 3 mg/kg *Q2W*	Ⅱ	84%	24%	10%	PD-L1, lymphocytes, TCR
mk3475-223	Ⅰ-Ⅱ	Pembrolizumab 200 mg *Q3W*	Ⅰ	87%	31%	15%	PD-L1
LCMC3	Ⅰb-Ⅲb	Atezolizumab 1, 200 mg *Q3W*	Ⅱ	88%	21%	7%	PD-L1, TMB, lymphocytes, exome sequence
Forde *et al*	Ⅰb-Ⅲa	Nivolumab 3 mg/kg *Q2W*	Ⅱ	95%	45%	15%	PD-L1, TMB, lymphocytes, TCR
Li *et al*	Ⅰb-Ⅲa	Sintilimab 200 mg *Q3W*	Ⅱ	93%	41%	16%	PD-L1
NEOSTAR	Ⅰ-Ⅲa	Nivolumab 3 mg/kg *Q2W* Iplilimumab 1 mg/kg d1	Ⅱ	84%	50%	38%	PD-L1, lymphocytes, TCR
NADIM	Ⅲa	Nivolumab 360 mg/kg *Q3W* Paclitaxel plus platinum	Ⅱ	89%	82.9%	63%	PD-L1, TMB, TIL, TCR/RNA-seq, ctDNA, MRD
Shu *et al*	Ib-Ⅲa	Atezolizumab 200 mg/kg *Q3W* Paclitaxel plus platinum	Ⅱ	79%	50%	21%	PD-L1
NSCLC: non-small cell lung cancer; MPR: major pathological rate; pCR: pathologic complete response; PD-L1: programmed cell death ligand 1; TCR: T cell receptor; TMB: tumor mutational burden; TIL: tumor infiltrating lymphocyte; ctDNA: circulating tumor DNA; MDR: minimal residual disease.							

**表 2:** 正在进行的可切除NSCLC新辅助免疫治疗Ⅲ期临床试验 Ongoing phase Ⅲ clinical trials of neoadjuvant immunotherapy in operable NSCLC

	Clinical Trial	NCT	Study design	*n*	Stage	Drugs	Primary endpoint
Imported drugs	CA209-77T	NCT04025879	Random Double-blind	452	Ⅱa-Ⅲb (T3N2)	Nivolumab plus chemotherapy or placebo	EFS
	CheckMate 816	NCT02998528	Random open	350	Ⅰb (T > 4 cm)-Ⅲa	Nivolumab plus chemotherapy or placebo	pCR, EFS
	KEYNOTE-671	NCT03425643	Random Double-blind	786	Ⅱ-Ⅲb	Pembrolizumab plus chemotherapy or placebo	EFS, OS
	IMpower030	NCT03456063	Random Double-blind	374	Ⅱ-Ⅲb (T3N2)	Atezolizumab plus chemotherapy or placebo	MPR, EFS
	AEGEAN	NCT03800134	Random Double-blind	300	Ⅱa-Ⅲb (N2)	Durvalumab plus chemotherapy or placebo	MPR
Domestic drugs	RATIONALE 315	NCT04379635	Random Double-blind	380	Ⅱ-Ⅲa	Tislelizumab plus chemotherapy or placebo	MPR, EFS
	JS001-029-Ⅲ-NSCLC	NCT04158440	Random Double-blind	406	Ⅲa	Toripalimab plus chemotherapy or placebo	MPR
EFS: event free survival; OS: overall survival.							

**表 3:** 新辅助免疫治疗生物标志物汇总 Summary of biomarkers for neoadjuvant immunotherapy

Tumor cell associated biomarkers	TME-related biomarkers	Liquid biopsy-related biomarkers	Host-related markers
PD-L1	Tumor-infiltrating immune cells	ctDNA	Enterococcus
TMB	With specific phenotypes: CD4^+^ T and CD8^+^ T cells; CD68^+^ macrophages; PD-1^+^ lymphocytes; cytotoxic T cell: CD3^+^CD8^+^; T memory cell: CD45RO^+^; resident memory T cell: CD8^+^CD103^+^; regulatory T cell: FOXP3^+^ CD4^+^PD-1^+^ T cells; CD3^+^PD-1^+^ T cells	Peripheral blood cells	Akarnania
Specific mutants *STK11*/*EGFR*/A*LK*, *et al*		NLR, M: L ratio PLR variation	
	With diversity of immune repertoires richness and clonality of TCR	Immune cell subtypes	
	Neoantigen specific TIL	TCR repertoire	
PD-1: programmed cell death-1; EGFR: epidermal growth factor receptor; ALK: anaplastic lymphoma kinase; NLR: neutrophil to lymphocyte ratio; M: L: myeloid cells: lymphocyte.			

2018年Forde等^[7]^在《新英格兰医学杂志》（*N Engl J Med*）上首次报道了在22例可手术NSCLC患者中进行的纳武利尤单抗新辅助治疗Ⅱ期临床试验（CheckMate159）。其结果显示20例完成新辅助免疫治疗的患者成功实施R0（显微镜下无残留）切除，手术患者的客观缓解率（objective response rate, ORR）仅为10%，但MPR率达到45%（95%CI: 23%-68%），pCR率为15%。CheckMate159研究作为先锋研究开启纳武利尤单抗新辅助治疗在早期NSCLC人群的疗效探索，同时发现抗PD-1治疗后诱导了外周血中突变相关新抗原特异性T细胞克隆的扩增。

2021年Provencio等^[8]^在世界肺癌大会（World Conference on Lung Cancer, WCLC）上发布最新数据显示46例可切除Ⅲa期NSCLC接受纳武利尤单抗联合含铂化疗新辅助治疗的Ⅱ期临床试验中（NADIM），41例患者接受手术治疗，均为R0切除。术后患者ORR为76%，MPR率为82.9%，pCR率为63%，3年无病生存（disease-free survival, DFS）率为69.6%（95%CI: 54.1-80.7%），3年OS率为81.9%（95%CI: 66.8-90.6%）。研究结果提示新辅助纳武利尤单抗联合化疗在Ⅲa期NSCLC人群中进一步带来病理缓解和生存获益。NADIM研究基于组织学标本和血液标本进行多种生物标志物与疗效预测的相关性探索。

2021年Cascone等^[9]^在《自然医学杂志》（*Nat Med*）上发表了44例可手术的NSCLC患者接受纳武利尤单抗或者联合伊匹木单抗新辅助治疗的Ⅱ期随机临床试验（NEOSTAR），结果显示接受手术R0切除的37例患者中，纳武利尤单抗单药组及纳武利尤单抗和伊匹木单抗联合治疗组MPR率分别为24%（5/21）和50%（8/16）。pCR率分别为10%和38%。术后单药组患者的ORR为22%，联合组为19%。中位OS和中位无复发生存期（relapse-free survival, RFS）均未达到。该研究因毒副作用提前终止，但pCR的患者获得长期无病生存，提示需要进一步探索预测新辅助纳武利尤单抗联合化疗的疗效标志物。

2021年Forde等^[10]^在美国癌症研究协会（American Association for Cancer Research, AACR）国际会议上发表的358例可手术切除的NSCLC患者纳武利尤单抗联合含铂双药化疗或者联合伊匹木单抗组对比含铂化疗的新辅助Ⅲ期随机对照研究（CheckMate816），结果显示纳武利尤单抗联合含铂双药化疗对比含铂双药化疗新辅助治疗后，接受手术R0切除的比例为83% *vs* 75%，ORR为54% *vs* 37%，MPR为36.9% *vs* 8.9%，pCR为24.0% *vs* 2.2%。研究还提示ctDNA的清除率可以预测新辅助纳武利尤单抗联合化疗的疗效。

目前仍有多项正在进行的Ⅲ期随机对照临床研究（表 2），将免疫联合化疗作为常用的试验组方案，这将为免疫治疗的新辅助用药模式提供重要证据。

## 2 新辅助免疫治疗生物标志物的研究

早期NSCLC中的生物标志物研究与晚期NSCLC中生物标志物的研究大致相似，从检测的标本类型上主要集中在基于组织学检测肿瘤和肿瘤微环境相关的生物标志物和基于外周血标本检测以及涉及宿主相关的标志物研究。

### 2.1 肿瘤细胞相关的生物标志物

#### 2.1.1 PD-L1和TMB

对于在晚期NSCLC研究比较成熟的生物标志物，如PD-L1、TMB。在早期NSCLC人群中，基于肿瘤细胞和/或免疫细胞上PD-L1的表达与主要病理缓解MPR或生存获益的相关性研究正在进行中。

CheckMate159研究^[7]^纳入了21例接受纳武利尤单抗新辅助治疗的可切除NSCLC患者。结果提示，初诊时无论肿瘤细胞PD-L1表达如何，均观察到肿瘤MPR。其中1例标本接受ICIs新辅助治疗后肿瘤细胞的PD-L1由阴性转为阳性。

LCMC3（NCT02927301）研究^[11]^中，纳入181例可切除的NSCLC患者，患者在接受2个周期阿替利珠单抗新辅助治疗后，在所有患者中均观察到MPR，初诊时肿瘤标本PD-L1水平分层，发现PD-L1≥50%组MPR率为33%，PD-L1 < 50%组MPR率为13%，且两组具有统计学差异。并未发现基线/手术时TMB与MPR的相关性，进一步研究TMB的cutoff值为10或者16，也未发现TMB与MPR相关性。

在NADIM研究^[8]^中，可切除NSCLC患者接受2个周期的纳武利尤单抗联合化疗新辅助治疗，获得完全病理缓解者，基线时肿瘤活检PD-L1表达更高。但未观察到PD-L1表达或者TMB与长期生存[无进展生存期（progression-free survival, PFS）PFS/ OS]获益相关。

PD-L1的预测价值有待进一步的数据进行验证。《肿瘤突变负荷应用于肺癌免疫治疗的专家共识》暂不推荐TMB用于预测免疫新辅助治疗疗效^[12]^。

#### 2.1.2 驱动基因突变

驱动基因突变在晚期NSCLC人群中对免疫治疗的预测作用包括正向预测和负向预测。其中表皮生长因子受体（epidermal growth factor receptor, *EGFR*）突变和间变性淋巴瘤激酶（anaplastic lymphoma kinase, *ALK*）融合对靶向治疗应答较好，对于免疫治疗的应答较差。*KRAS*突变和*BRAF* V600E突变NSCLC对免疫治疗可能获得较好疗效。在早期的NSCLC人群中驱动基因对于免疫治疗应答的报道数据有限。在LCMC3研究^[12]^中，基线*SKT11*突变型的患者较野生型患者获得病理缓解率较差。NADIM研究^[8]^中，驱动基因*EGFR*/*STK11*/*KEAP1*/*RB1*突变的患者较野生型患者的无进展生存期PFS更短，提示该突变类患者更难从新辅助免疫联合化疗中获益。

### 2.2 肿瘤微环境相关的生物标志物

新辅助治疗模式较其他晚期NSCLC人群的治疗模式相比，其独特优势在于可同时获取新辅助治疗前组织标本和治疗后手术大标本，通过组织学染色/基因组检测可以直观地对比肿瘤微环境中相关免疫细胞对免疫药物应答的不同反应。

肿瘤的免疫应答以细胞免疫为主，其中T淋巴细胞在肿瘤免疫中起着核心调控作用，其细胞的功能取决于T淋巴细胞总值CD3^+^（完全成熟的T细胞）及其亚群（辅助T淋巴细胞CD4^+^、细胞毒性T细胞CD8^+^）的相对组成来维持体内免疫系统的最佳平衡。早期披露NSCLC人群中，CD4^+^和CD8^+^ T细胞大量浸润与肿瘤良好生存预后相关^[13]^。

新辅助治疗后获得MPR/pCR的患者，术后切除标本肿瘤微环境中免疫细胞表型较新辅助免疫治疗前免疫细胞表型发生变化。CheckMate159研究中获得MPR者，治疗前活检标本瘤内只含有少量PD-L1^+^巨噬细胞，新辅助纳武利尤单抗治疗后，瘤内浸润大量CD8^+^阳性和PD-1阳性的免疫细胞，以及少部分浸润免疫细胞PD-L1表达水平升高。值得注意的是，获得pCR的切除标本中，视野中大量浸润CD8^+^ T细胞、PD-1^+^淋巴细胞、CD68^+^巨噬细胞，出现FoxP3^+^调节性T细胞及三级淋巴结构^[7]^。

在NADIM研究^[8]^中，对比未获得主要病理缓解（incomplete pathologic response, IPR）即肿瘤细胞所在区域小于90%坏死，获得pCR即肿瘤细胞100%坏死和MPR即肿瘤细胞90%坏死，有较高水平的记忆T细胞（CD45RO^+^）和调节T细胞（FOXP3^+^）以及巨噬细胞（CD68^+^）产生。其他研究数^[14]^据也显示肿瘤组织驻留的记忆T细胞（CD8^+^CD103^+^）富集与早期NSCLC的良好预后相关。在NEOSTAR研究^[15]^中，新辅助纳武利尤单抗联合伊匹木单抗治疗后，患者手术标本中有更高水平的CD3^+^ TILs和更高水平的组织驻留记忆T细胞（tissue-resident memory, TRM）。

肿瘤微环境中不同表型的淋巴细胞，如CD4^+^PD-1^+^ T细胞和CD3^+^PD-1^+^ T细胞的富集对新辅助免疫治疗疗效有一定预测作用。NADIM研究中获得pCR的标本中含有较高水平的CD4^+^PD-1^+^ T细胞，其作为预测标志物的受试者操作特征曲线（receiver operating characteristic, ROC）的曲线下面积（area under the curve, AUC）值为0.728。同样，在LCMC3（NCT02927301）研究中，对比未获得MPR者，获得MPR者CD3^+^PD-1^+^ T细胞出现富集^[16]^，其AUC值为0.72。进一步的研究基于肿瘤微环境中多个免疫细胞亚型RNA测序分析预测新辅助免疫治疗的疗效。在LCMC3研究^[16]^中，对手术切除标本进行单细胞测序，结果提示，ILT2的表达与MPR呈正相关，多表达于树突细胞、单核细胞和巨噬细胞，且与PD-L1表达呈线性相关，提示LIT2与PD-L1在同一细胞上共表达。

上述研究数据表明肿瘤微环境中肿瘤浸润免疫细胞的不同表型对新辅助免疫治疗应答具有一定预测作用，其他的生物标志物仍需继续探索。

免疫组库的多样性和克隆性与疗效相关性的研究^[17]^表明肿瘤组织中的TCR库可能可以预测可切除Ⅲa（N2）期NSCLC患者接受新辅助序贯化疗-度伐利尤单抗免疫治疗的复发风险，治疗后肿瘤组织TCR库与无事件生存率（event free survival, EFS）、MPR、结节清除率相关。NEOSTAR研究中，与基线诊断时的肿瘤相比，切除的肿瘤标本中TCR库组成的多样性及反应性增加；对比癌旁肺组织，切除肿瘤的TCR库丰富度和克隆型在新辅助单药和联合组均显著增加。

单细胞转录组学揭示了TIL在免疫应答过程中功能发生障碍，从而使得大多数TIL不能识别肿瘤进行杀伤，新抗原特异性TIL转录组测序获益于新辅助治疗模式组织标本的获得。NADIM研究近期发表在*Nature*杂志的数据^[18]^表明在MPR和非MPR患者的手术切除标本中进行突变相关新抗原特异性TIL的RNA测序发现两组转录谱的表达显著不同。在非MPR的突变相关新抗原特异性TIL中，T细胞耗竭相关的基因（*TOX2*、*CTLA4*、*HAVCR2*、*ENTPD1*）水平显著升高，在MPR的突变相关新抗原特异性TIL中，记忆T细胞[白介素7受体（interleukin 7 receptor, IL-7R）、转录因子7（transcription factor 7, TCF7）]和效应T细胞（GZMK）相关的基因表达水平升高。与来自肿瘤治疗应答的细胞克隆相比，来自无应答的突变相关新抗原特异性T细胞（主要是转录因子HOBBIT高表达的组织驻留记忆T细胞亚群）其配体依赖的TCR信号传导明显降低，并上调免疫检查点和杀伤抑制性受体表达及T细胞活化的抑制分子^[18]^。这些发现为克服对PD-1阻断剂的耐药性提供了重要线索。

肿瘤微环境中相关生物标志物的发掘首先研究包括肿瘤浸润免疫细胞表型在新辅助治疗前后发生的变化。如CD4^+^和CD8^+^ T细胞、CD68^+^巨噬细胞、PD-1^+^淋巴细胞以及不同状态的TIL，其中CD4^+^PD-1^+^ T细胞和CD3^+^PD-1^+^ T细胞对于新辅助免疫治疗药物的疗效具有一定的预测作用。其次，包括免疫组库的多样性研究，如肿瘤组织标本TCR库的克隆性和多样性检测。最后，基因组学的其他标志物仍在探索中，如新抗原特异性TIL的转录谱与MPR的相关性。

### 2.3 外周血相关的生物标志物

外周血标本与组织标本相比具有非侵袭性、重复采样风险低、获取便捷等优点，同时，外周血可以提供宿主全面的免疫状态。因此，有研究^[19]^已经探索了基于外周血的抗PD-（L）1治疗反应的潜在预测因子，例如ctDNA、血细胞计数、循环免疫细胞以及基于外周标本检测的T细胞受体等。

#### 2.3.1 外周血中的ctDNA

基于外周血中的ctDNA的测序可以应用于分子残留病灶（molecular residual disease, MRD）检测，可辅助识别术后复发高风险的患者。一项大规模、前瞻性多队列研究纳入330例Ⅰ期-Ⅲ期NSCLC患者，采用二代测序（next generation sequencing, NGS）检测血浆标本并用组织标本进行验证^[20]^。结果发现术后1个月内ctDNA-MRD阳性患者的复发率为80.8%（21/26），显著高于阴性患者的16.2%（49/303）（*P* < 0.001）；多因素*Cox*分析显示术后ctDNA-MRD阳性是患者无复发生存期（relapse free survival, RFS）缩短的独立危险因素（*P* < 0.001）^[19]^。

ctDNA的清除与免疫治疗疗效的相关性研究正在进行中。CheckMate816研究^[10]^中，在第1周期以及第3周期的第1天分别采集外周血标本进行ctDNA检测。实验组有56%（24/43）的患者出现ctDNA清除现象，化疗组为34%（15/44）。其中，实验组ctDNA清除的患者中46%（11例）获得pCR，而ctDNA未清除的患者无pCR。化疗组出现ctDNA清除的患者13%（2例）获得pCR，ctDNA未清除的患者有3%（1例）获得pCR。该结果一方面提示新辅助免疫联合化疗在ctDNA清除上优于化疗，另一方面提示ctDNA清除与pCR之间具有某种程度的一致性。NADIM研究^[8]^中，NGS分析43例治疗前血浆样本中有30例基线时检测到ctDNA，多变量分析显示，ctDNA的清除可以显著预测长期生存（PFS: HR=0.3, *P*=0.072; OS: HR=0.05, *P*=0.024）。

#### 2.3.2 外周血细胞计数和外周免疫细胞

常规抽血的血细胞计数及比值可以作为肿瘤对ICIs反应的潜在标志物^[20]^。在晚期NSCLC中，血液中较高的中性粒细胞与淋巴细胞之比（neutrophil and lymphocyte ratio, NLR）和较高的髓系细胞与淋巴细胞之比（myeloid cells: lymphocyte, M: L）与PD-1治疗的不良的生存预后相关^[21]^。NADIM研究^[8]^中发现，新辅助免疫联合化疗后，外周血中白细胞、嗜酸性粒细胞、单核细胞、嗜中性粒细胞、血红蛋白总体水平均下降。但是，淋巴细胞、嗜碱性粒细胞和乳酸脱氢酶（lactate dehydrogenase, LDH）水平没有发生变化。新辅助免疫治疗后外周血NLR和M: L的比值以及血小板与淋巴细胞之比（platelet lymphocyte ratio, PLR）降低。对比未完全缓解的肿瘤（incomplete pathologic response, IPR）患者，pCR患者血液中PLR的变化显著降低。但尚需要进一步大样本量的试验验证血细胞作为新辅助免疫治疗潜在的生物标志物的预测效果。

外周血免疫表型对MPR预测作用的探索。LCMC3研究^[16]^中通过10色60标记的流式细胞术评估基线状态下外周血的综合免疫表型，结果表明，外周血中表达ILT2和NKG2A的自然杀伤（natural killer, NK）细胞和NK样T细胞等固有免疫细胞与新辅助免疫治疗的疗效应答有关。

#### 2.3.3 TCR测序

免疫系统发挥抗肿瘤作用需要过克隆增殖的TCR来识别新抗原肽，TCR的克隆性和多样性可以通过深度测序来评估。外周血TCR库作为免疫治疗应答的标志物研究才刚起步。

有研究^[7]^表明在早期肺腺癌中外周血中较高密度T细胞与OS获益相关。NADIM研究^[22]^中，基于外周血测序的TCR均一性、多样性、克隆性均不能预测新辅助ICIs联合治疗的疗效。但发现TCR常见克隆型升高与MPR和PFS相关。目前仍需要大规模的临床研究来验证外周血TCR作为新辅助ICIs生物标志物的预测效果。

### 2.4 肠道微生物

肠道共生菌群可调节宿主免疫系统和组织稳态，也是免疫治疗生物标志物研究的对象之一^[23]^。NEOSTAR研究中，采用16S V4rRNA基因序列分析治疗前后的粪便标本表明，ICIs对微生物群落的多样性、结构和组成没有明显影响。然而，定性的生物标志物研究^[8]^发现，双免疫疗法组中MPR的患者，治疗前的标本中肠道球菌和*Akkermansia*菌的丰富度较高。目前NADIM研究和其他的研究也在进行肠道微生物的探索。

## 3 小结

ICIs全面革新了NSCLC患者的治疗格局，为晚期NSCLC人群带来长期生存获益，在早期NSCLC人群的研究数据亦令人鼓舞。生物标志物的发现便于我们在临床实践中找到从ICIs新辅助治疗中真正获益的人群。

本文讨论了ICIs用于新辅助治疗早期可切除NSCLC人群的研究中基于肿瘤组织、血液、粪便的相关标志物对ICIs疗效的预测作用。肿瘤微环境相关生物标志物研究提示免疫治疗引起肿瘤微环境中免疫细胞相关指标发生变化。转录组分析发现，在非MPR者中，突变相关新抗原特异性TIL表现为T细胞耗竭相关的基因水平显著升高，而在MPR的突变相关新抗原特异性TIL表现为记忆T细胞和效应T细胞相关的基因表达水平升高。另一方面，初期数据提示CD4^+^PD-1^+^ T细胞或者CD3^+^PD-1^+^ T细胞的富集可能与免疫治疗的疗效相关。基于血液标本检测的不同标志物的研究数据初步披露，术后ctDNA-MRD阳性是患者RFS缩短的独立危险因素，同时发现ctDNA的清除可以显著预测长期生存。其他涉及肿瘤细胞相关或者宿主相关的标志物研究数据提示未观察到PD-L1表达或者TMB与患者的长期生存（PFS/OS）获益相关。肠道微生物与免疫治疗的疗效相关性是研究的热点，目前处于初期阶段。

未来需要开展前瞻性、大样本量的临床研究，进一步探索评价早期可切除NSCLC人群新辅助免疫治疗效果的生物标志物，最大化发挥ICIs对早期NSCLC患者的疗效，同时排除对ICIs可能存在不良反应的人群。
